# Primary small cell neuroendocrine carcinoma of the larynx: A detailed case report

**DOI:** 10.1097/MD.0000000000041574

**Published:** 2025-02-14

**Authors:** Siqi Mu, Xiaosong Wu

**Affiliations:** a Department of Otorhinolaryngology, Chongqing University Central Hospital, Chongqing Emergency Medical Center, The Fourth People’s Hospital of Chongqing, Chongqing, China.

**Keywords:** early detection, larynx, small cell neuroendocrine carcinoma, smoking, surgical intervention

## Abstract

**Rationale:**

Small cell neuroendocrine carcinoma (SCNEC) of the larynx is a rare and aggressive malignancy, notorious for its rapid progression and poor prognosis. Early diagnosis and effective management are crucial, particularly for high-risk populations such as long-term smokers.

**Patient concerns:**

A young male smoker presented with progressive laryngeal symptoms, which prompted further investigation due to the severity and progression of his symptoms.

**Diagnoses:**

The patient was diagnosed with SCNEC of the larynx after presenting with symptoms. Diagnostic procedures confirmed the presence of a localized tumor without distant metastasis.

**Interventions:**

In response to the diagnosis, the patient underwent a surgical intervention that achieved clear margins, chosen based on the tumor’s localized nature at diagnosis. This approach emphasized comprehensive initial surgical management.

**Outcomes:**

The absence of immediate postoperative recurrence highlights the potential effectiveness of the surgical approach as a standalone treatment in selected cases of SCNEC.

**Lessons:**

This case underscores the importance of considering SCNEC in the differential diagnosis when young smokers present with laryngeal symptoms. It highlights the need for proactive surgical strategies to manage this aggressive cancer effectively. Additionally, it emphasizes that despite the favorable initial outcome, the high risk of recurrence necessitates rigorous long-term follow-up and a structured surveillance strategy to enhance early detection and potentially improve long-term outcomes. This report offers significant insights into the rapid progression and nuanced management of SCNEC, providing valuable lessons for clinical practice.

## 1. Introduction

Small cell neuroendocrine carcinoma (SCNEC) of the larynx is a rare and highly malignant cancer. This report emphasizes the critical role of timely surgical intervention and the importance of early diagnosis in improving patient outcomes.

## 2. Case report

A male in his early 40s was admitted to the hospital presenting with progressive hoarseness that began a month prior without any obvious precipitating factors. The patient reported no associated symptoms such as sore throat, coughing, dysphagia, chest discomfort, or respiratory difficulties. He did not initially seek medical attention and had a history of smoking for over 20 years, consuming 10 to 20 cigarettes daily. His medical history was unremarkable for chronic diseases, surgical interventions, or traumatic injuries, and there was no family history of genetic disorders or malignancies.

**Figure 1.**
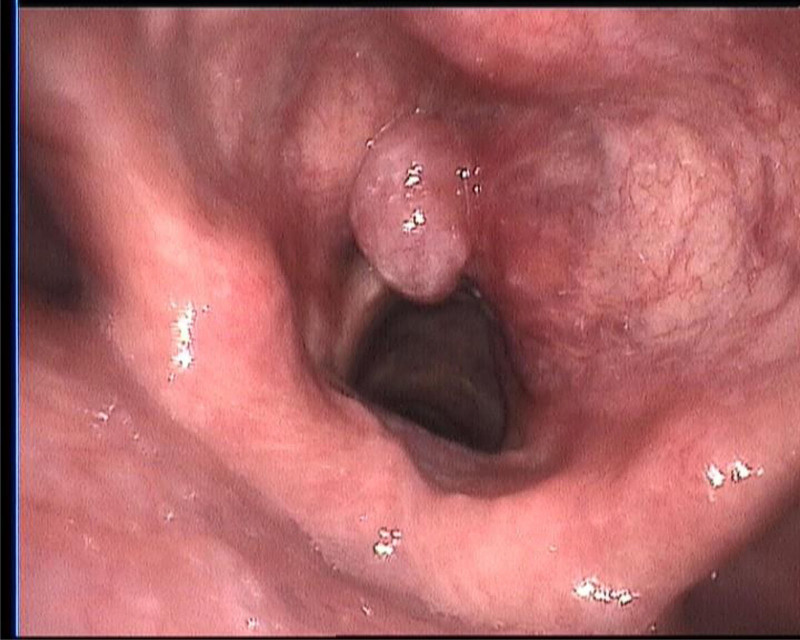
Preoperative electronic laryngoscopy. This image from an electronic laryngoscopy shows a smooth, well-defined nodular lesion on the right vocal cord, causing partial airway obstruction.

**Figure 2.**
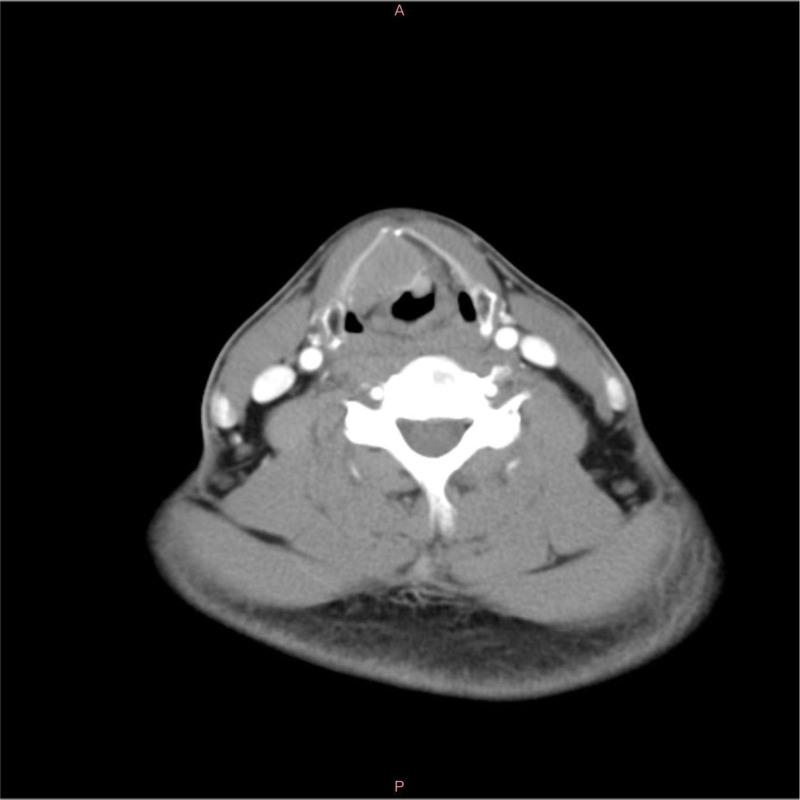
Axial computed tomography (CT) scans of the larynx. This CT scan at the level of the vocal cords displays a well-defined mass in the right paraglottic space. The mass demonstrates homogeneous enhancement and is adjacent to but not invading the thyroid cartilage.

Upon initial evaluation, an electronic laryngoscopy revealed a pale red, smooth neoplasm obstructing part of the vocal cords and obscuring the anterior commissure, affecting the mobility of the right vocal cord and impairing closure on both sides (Fig. 1). An enhanced neck computed tomography scan showed a mass involving the right vocal cord, anterior commissure, and adjacent paraglottic space, suggestive of a neoplastic lesion (Fig. 2). A biopsy performed during the hospital stay identified the tumor originating from the right vocal cord and extending into the right paraglottic space. Frozen section analysis confirmed the malignant nature of the tumor.

**Figure 3.**
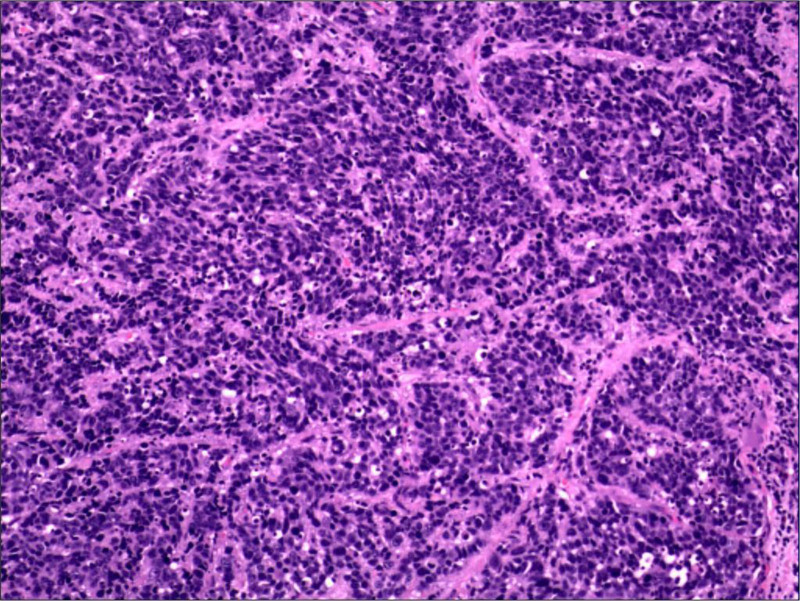
Postoperative pathological findings. This image shows a biopsy from the larynx stained with hematoxylin and eosin, displaying characteristic features of small cell neuroendocrine carcinoma. Notable is the densely packed small cells with high nuclear-to-cytoplasmic ratios and prominent mitotic figures, indicative of aggressive tumor behavior.

**Figure 4.**
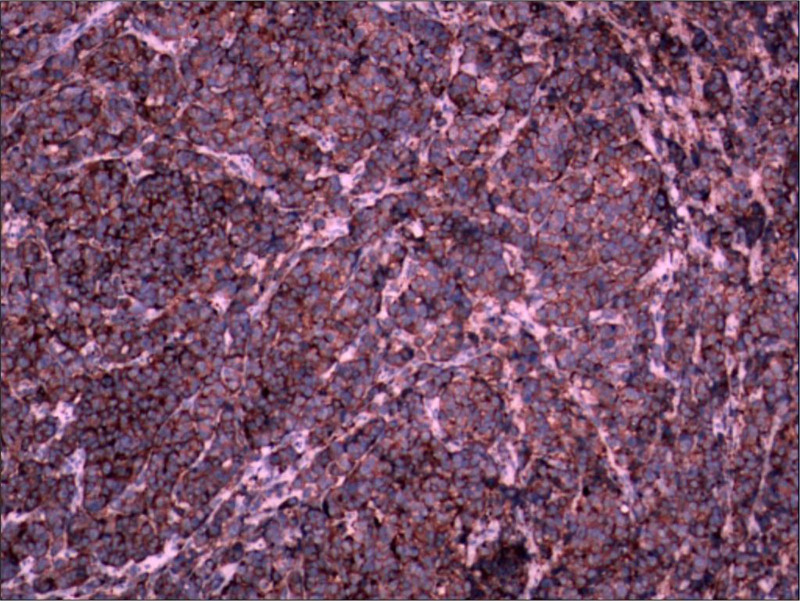
Postoperative pathological findings. Immunohistochemistry staining for synaptophysin in the tumor tissue shows strong, diffuse cytoplasmic positivity, confirming the neuroendocrine origin of the carcinoma.

The patient underwent a right vertical partial laryngectomy after comprehensive discussions with him and his family. Surgical margins were negative, and no lymph node metastasis was detected. Postoperative pathology confirmed the diagnosis of SCNEC of the larynx, supported by immunohistochemical findings: CD56(+), SYN(+), CgA(−), CK(focal+), CK5/6(−), CD45(−), Vimentin(−), p53(−), and Ki-67 proliferative index of 80% (Figs. 3 and 4). The final diagnosis was glottic cancer, and the clinical stage was T2N0M0. The patient was followed for 6 months postoperatively, showing no signs of local recurrence; however, he did not return for subsequent follow-ups. The high Ki-67 index indicated a high level of tumor aggressiveness, underscoring the necessity for diligent monitoring and potential adjuvant therapy to manage any recurrence or metastasis effectively.

## 3. Discussion

SCNEC of the larynx, although rare, demonstrates a highly malignant profile with rapid progression and poor prognosis, characteristics that significantly challenge effective clinical management. Key studies highlight that due to the nonspecific initial symptoms such as hoarseness or a sore throat, SCNEC is often misdiagnosed as less aggressive laryngeal pathologies, which leads to delays in appropriate treatment.^[1]^

Additionally, the rarity of the disease contributes to the lack of standardized treatment protocols, further complicating the management of patients^[2]^ in the early identification and precise histopathological classification necessary to tailor the aggressive treatment needed.^[3]^ Meanwhile, this case underscores the association between smoking and the aggressiveness of SCNEC, highlighting smoking as a significant etiological factor.^[4]^ By demonstrating the severe progression despite seemingly manageable early symptoms, this case supports the literature that advocates for heightened clinical vigilance and routine screening for high-risk individuals, particularly long-term smokers.^[5]^

## 4. Conclusion

This case of SCNEC in the larynx underscores the importance of considering this aggressive carcinoma in the differential diagnosis for young smokers presenting with laryngeal symptoms. The findings advocate for a proactive surgical approach with sufficient margins to manage the disease effectively, especially in patients without distant metastasis at presentation. The absence of immediate postoperative recurrence reinforces the potential viability of surgical management as a standalone initial treatment in selected cases. However, the aggressive nature of SCNEC and the high risk of recurrence necessitate long-term follow-up, emphasizing the need for a structured surveillance strategy to ensure early detection of recurrence, thereby potentially improving long-term outcomes. This report contributes significantly to the existing literature by providing a detailed analysis of the rapid progression and management of SCNEC, highlighting the critical elements that should inform future clinical practice.

## Acknowledgments

The authors extend their deepest gratitude to the patient and his family for their cooperation and consent to share this case, which contributes significantly to the medical community’s understanding of small cell neuroendocrine carcinoma of the larynx. The authors also thank the medical and surgical teams at Chongqing University Central Hospital, whose expertise and dedication were crucial in the management of this case. Special thanks to the pathology department for their expedient and thorough analyses, which were vital for the timely diagnosis and treatment planning. Additionally, the authors acknowledge the support staff for their invaluable assistance throughout the treatment and follow-up phases.

## Author contributions

**Writing—original draft:** Siqi Mu.

**Writing—review & editing:** Xiaosong Wu.
